# Higher Risk, Higher Reward? Self‐Reported Effects of Real‐World Cannabis Use in Parkinson's Disease

**DOI:** 10.1002/mdc3.13414

**Published:** 2022-01-28

**Authors:** Samantha K. Holden, Christopher H. Domen, Stefan Sillau, Ying Liu, Maureen A. Leehey

**Affiliations:** ^1^ Department of Neurology University of Colorado Anschutz Medical Campus Aurora Colorado USA; ^2^ Department of Neurosurgery University of Colorado Anschutz Medical Campus Aurora Colorado USA

**Keywords:** Parkinson's disease, cannabis, cannabidiol, 9‐Δ‐tetrahydrocannabinol, therapy

## Abstract

**Background:**

Despite limited evidence, people with Parkinson's disease (PD) use cannabis for therapeutic purposes. Given barriers to performing randomized trials, exploring real‐world experiences with cannabis in PD is valuable.

**Objective:**

Investigate the frequency and magnitude of symptomatic effects reported with cannabis use in PD.

**Methods:**

An anonymous, 15‐question, web‐based survey was deployed on Fox Insight. Cannabis product types were defined (by relative tetrahydrocannabinol [THC] and cannabidiol [CBD] content) and respondents were asked to reference product labels. Questions focused on use patterns and subjective effects on 36 predefined symptoms (rated −2‐markedly worse to +2‐markedly better).

**Results:**

1,881 people with PD responded (58.5% men; mean age 66.5; 50.5% <3 years of PD). 73.0% of respondents reported medicinal use, though 30.8% did not inform their doctor. 86.7% knew their type of cannabis product: 54.6% took higher CBD, 30.2% higher THC, and 15.2% took similar amounts of THC and CBD products. Most common use was oral administration, once daily, for less than six months. Frequent improvements were reported for pain, anxiety, agitation, and sleep (>50% of respondents, mean magnitude 1.28–1.51). Dry mouth, dizziness, and cognitive changes were common adverse effects (20.9%–30.8%, mean −1.13 to −1.21). Higher THC users reported more frequent improvements in depression, anxiety, and tremor, and more frequent worsening in dry mouth and bradykinesia than other product types.

**Conclusions:**

Respondents with PD reported using more CBD products, via oral administration, with mild subjective benefits primarily for sleep, pain, and mood. Higher THC products may be higher risk/higher reward for PD‐related symptoms.

Cannabis use increased by approximately 1000% between 2007 and 2016 among adults over the age of 65.^1^ For people with Parkinson's disease (PD), 36.6% endorsed using cannabis for symptom management in a recent survey^2^; 72.3% described their use as medicinal even though there is little evidence supporting benefit. Questions regarding the medicinal utility of cannabis may be attributable to the complexity of the plant, as it contains over 100 cannabinoids, the two most well‐known of which are Δ9‐tetrahydocannabinol (THC) and cannabidiol (CBD). THC has intoxicating and analgesic properties, whereas CBD is not intoxicating but may also have analgesic and anti‐inflammatory properties.^3^ Unfortunately, THC may induce anxiety,^4^, ^5^, ^6^ cognitive dysfunction,^4^, ^5^, ^6^, ^7^, ^8^, ^9^ imbalance,^8^, ^10^ and hallucinations,^6^, ^11^ and has potential to cause serious adverse effects in medically fragile people with PD. Alternatively, studies suggest that CBD reduces psychosis^12^, ^13^ and anxiety.^14^, ^15^ However, a recent open label study^16^ found that high doses of CBD led to adverse effects in all participants with PD and that one‐third developed abnormalities in liver enzymes.

There is a wide variety of THC and CBD cannabis products available to consumers in states with legalized cannabis. In addition, hemp products, which are made from a cannabis plant that is high in CBD and have <0.3% THC, are widely available. At present, choosing a specific cannabis product is mostly self‐guided for people with PD given a lack of evidence‐based guidelines. The ratio and individual dosages of cannabinoids, as well as route of administration, may impact symptomatic effects of cannabis products.^11^ A dose‐response relationship between THC and its effects has been observed for some symptoms, including cognition,^17^ while biphasic responses (i.e., differential responses at low versus high doses) have been observed for other symptoms, including anxiety.^18^ There is also likely a synergistic relationship between CBD and THC,^19^ with CBD perhaps enhancing the effects of THC. Further complicating matters, oral administration of THC‐laden cannabis versus combustion results in delayed onset and longer lasting symptoms of intoxication, related to the time it takes for THC to be absorbed by the intestine and transported to the liver.^20^ To the greatest extent possible, we must improve our understanding of the effects of specific doses and types of cannabis to better inform therapeutic recommendations and clinical trial design.

From a pragmatic standpoint, surveying people with PD who have used cannabis could provide valuable information. Leveraging the reach of the Fox Insight survey platform through the Michael J. Fox Foundation, we aimed to better understand real‐world patterns of cannabis use among people with PD and to determine the frequency and magnitude of self‐reported symptomatic effects, both beneficial and adverse, with various types of cannabis products. With this knowledge, clinicians could more effectively counsel people with PD regarding potential effects of cannabis use and researchers could better design future clinical trials to more objectively study cannabis in PD.

## Methods

### Study Design and Participants

Fox Insight, sponsored by the Michael J. Fox Foundation, is an ongoing online clinical study establishing a large cohort of people with and without PD (current total n = 53,328), collecting self‐reported clinical variables. Data collection, management, and validation in Fox Insight are described by Smolensky and colleagues.^21^ Fox Insight participants are aged 18 or older and are recruited via electronic means, (e.g., social network ads or e‐newsletters). Participants fill out a standardized battery of questionnaires periodically and are also offered one‐time questionnaires on specific topics.

For this cross‐sectional observational study, all Fox Insight participants with PD received an email invitation to complete our survey (Supplementary Text A). Inclusion criteria required personal use of any cannabis product since onset of PD. Before opening the survey, respondents were required to acknowledge a Certificate of Confidentiality, which explained their risks and protections regarding their survey answers, as the legal status of cannabis varies by state. This constituted informed consent. If respondents declined, the survey was terminated. No respondents who agreed to the Certificate of Confidentiality were excluded from analyses.

### Survey Development

The survey introduction provided lay language definitions of cannabis, marijuana, THC, CBD, and hemp to ensure that participants could best identify which type of cannabis they had personally used (Supplementary Text B1). Survey questions were developed based on clinical experience with cannabis use in PD (SKH, ML) and multiple sclerosis (CHD),^22^ with technical guidance by the Fox Insight team. A beta version of the survey was administered to four people with PD on the Michael J. Fox Foundation Patient Council; changes were made based on their feedback to improve clarity of prompts and decrease respondent burden. The final survey (Supplementary Text B2) consisted of 15 multiple choice questions, with one item asking respondents to rate the effect of cannabis use on 36 PD‐related symptoms using a 5‐point Likert scale (−2 markedly worse to +2 markedly better). They could also report that they did not have a particular symptom or that the symptom started with cannabis use. Descriptions of each symptom were provided for clarity. Time required to complete the survey was approximately 15 min.

### Sample Size

Our sampling strategy focused on collecting useful data on the four major types of cannabis products: (1) high CBD/low THC; (2) high THC/low CBD; (3) similar amounts of THC and CBD; and (4) hemp. Based on our clinical experience, we predicted that the proportion of respondents with PD using each cannabis type would vary, estimating about 35% would use hemp, 30% high CBD/low THC, 25% similar amounts of THC and CBD, and 10% high THC/low CBD. A sample size of 200 respondents per cannabis product type group would allow, under the condition of maximum variance, detection of proportion differences of 0.16 with 90% power for categorical variables and differences in means of 0.33 times the within group standard deviation with 90% power for continuous variables. Thus, estimating that 10% of users would be taking high THC/low CBD, at least 2000 respondents were required. Conservatively, we estimated that 20% of Fox Insight participants with PD use cannabis and about 60% of them would complete the survey. Thus, overall response rate would be 12%, requiring a potential respondent pool of at least 16,667 people with PD. At the time of survey deployment, there were approximately 28,000 people with PD registered with Fox Insight.

### Statistical Analyses

Demographic and clinical characteristics of survey respondents were analyzed using descriptive statistics. Association tests for cannabis use characteristics by the four cannabis product type subgroups were performed using chi‐square, Fisher's exact, or ordinal correlation tests for categorical variables, and with ANOVA type, Kruskal Wallis, or correlation tests for continuous outcomes and scales. For individual survey items, frequencies and means, with 95% confidence intervals, of each response were tabulated for the overall cohort, then also for cannabis product type subgroups. For the symptomatic effect question, the frequency of each response was calculated from the Likert scale, only for those respondents who reported presence of that symptom prior to initiating cannabis use. Clinically meaningful frequencies of beneficial and adverse effects were defined as greater than 25% and 5%, respectively. To determine the magnitude of improvement or worsening for each symptom (from mild to marked), means were calculated for those respondents who reported any improvement (+1 or +2 on Likert scale) or any worsening (−1 or −2). These analyses were performed first for the overall cohort, then also by each of the four cannabis product type subgroups. These analyses were repeated for collapsed cannabis product type subgroups: (1) “higher THC” (high THC/low CBD); (2) “similar THC/CBD” (similar amounts of THC and CBD); and (3) “higher CBD” (included high CBD/low THC and hemp groups). To explore dose‐dependent responses within cannabis types, analyses were also performed by dosage, with high THC dose considered to be >50 mg and high CBD dose >200 mg.

To ensure generalizability of results, comparisons of demographic and clinical characteristics between our respondent group and the broader Fox Insight population with PD who were invited but did not open the survey were performed. Comparisons were also made between the group who completed the entire survey and those who opened the survey but declined to agree to the Certificate of Confidentiality to evaluate for response biases. Statistical significance was set at *P* = 0.05. Statistical analyses were performed using the SAS statistical software package (version 9.4; SAS Institute Inc., Cary, NC).

## Results

### Demographic and Clinical Characteristics of Respondents

The survey was deployed on the Fox Insight platform on 1/23/2020 and closed on 6/9/2020, with 1881 people completing the survey. Respondents were an average age of 66.5 ± 9.1 years old, were 58.5% male, and 97.9% white (Table 1). Most were in early stages of PD (50.5% with <3 y duration) and highly educated (64.0% with at least a college degree and 33.6% with graduate or professional degrees). An additional 627 people opened the survey but declined to agree to the Certificate of Confidentiality. This group was significantly more likely to choose “prefer not to answer” for income than those who completed the survey (16.8% who declined vs. 10.7% who agreed, *χ*2 = 17.6 [df = 3], *P* < 0.001) and less likely to identify as Native American for race (0.2% vs. 1.1%, *χ* ^2^ = 5.0 [df = 1], *P* = 0.03). In comparison to respondents, the overall PD population of Fox Insight participants who did not complete the survey (n = 32,464), was slightly older (67.2 years, *T* = 4.3, *P* < 0.0001), less male (55.5%, *χ* ^2^ = 6.4 [df = 1], *P* = 0.01), in later PD stages (44.5% <3 y disease duration, *χ* ^2^ = 45.4 [df = 3], *P* < 0.0001), and less likely to be college educated (59.8%, *χ* ^2^ = 13.3 [df = 1], *P* < 0.0001).

**TABLE 1:** Survey respondent demographics and clinical characteristics

	All respondents (n = 1881)
Age (yr)	66.5 (9.1)
Sex (% male)	58.5%
Parkinson's disease duration	
Early (<3 yr)	50.5%
Mid (3–10 yr)	37.9%
Late (>10 yr)	11.6%
Race	
White	97.9%
Black	0.7%
Native American	1.1%
Asian/Pacific Islander	1.2%
Ethnicity (% Hispanic)	3.4%
Education	
HS/GED or less	8.1%
Some College or Bachelor's/Associate's Degree	58.3%
Graduate/professional	33.6%
Income	
<$50,000/yr	25.9%
$50–100,000/yr	31.4%
>$100,000/yr	32.0%
Prefer not to answer	10.7%
Employment	
Full/part‐time	23.3%
Retired	70.3%
Unemployed	6.4%
Primary purpose of cannabis use	
Medicinal	73.0%
Recreational	7.3%
Both	19.7%
Type of cannabis product used	
High THC/Low CBD	26.2%
High CBD/Low THC	30.4%
Similar THC/CBD	13.1%
Hemp	16.9%
Do not know	13.3%
Method of cannabis use	
Smoke/Vape Only	20.5%
Smoke/Vape + Other	17.0%
Other:	62.5%
	–
Edible oil	30.4%
Food	29.3%
Sublingual tincture	24.5%
Skin cream	15.6%
Drink	3.4%
Skin patch	0.6%
Suppository	0.05%
Frequency of cannabis use	
>3 times/day	3.40%
2–3 times/day	19.1%
Daily	30.6%
Daily to weekly	22.3%
Weekly to monthly	9.5%
Less than monthly	15.2%
Duration of cannabis use	
>1 yr	33.0%
7–12 mo	14.8%
1–6 mo	30.7%
< 1 mo	21.5%
Discussion of cannabis use with doctor	
Yes	69.2%
No	31.8%

### Characteristics of Cannabis Use among Respondents

Respondents reported using cannabis primarily for medicinal purposes (73.0%), with broad variability in usage patterns (Table 1). Most respondents reported short duration of cannabis use (52.5% ≤6 months) at the time of answering the survey, though 33.0% reported greater than one‐year duration of use. Once daily was the most common frequency of use. Edible administration was most common, via food, drink, edible oil, or sublingual tincture, though 37.5% of respondents reported smoking or vaping their cannabis product. 31.8% of respondents reported not informing their doctor about their cannabis use.

86.7% of respondents could report the specific cannabis product type they were taking (high THC/low CBD, high CBD/low THC, similar THC and CBD, or hemp). Approximately two‐thirds of respondents were able to report exact THC (65.1%) and CBD (68.8%) dosages from their product labels. For THC, 23% of respondents reported taking <5 mg, 20.1% 6–50 mg, and 3.8% more than 50 mg per day (18.1% endorsed using products with no THC). For CBD, 21.7% were taking <5 mg, 24.7% 6–50 mg, 5.3% 51–200 mg, 2.0% 201–600 mg, and 2.3% >600 mg (12.9% endorsed using products with no CBD).

Grouping respondents by cannabis product types, “higher CBD” and “similar THC/CBD” product users were more likely to report primary medicinal purpose of cannabis use (89.9%) than “higher THC” users (46.9%, *P* < .0001). “Higher CBD” and “similar THC/CBD” product users were also less likely to smoke or vape, reporting non‐combustion methods of administration (oral, transdermal, suppository) at higher rates than combustion (87.2% vs. 28.8% respectively, *P* < .0001).

### Reported Symptomatic Effects with Cannabis Use

**FIG. 1:**
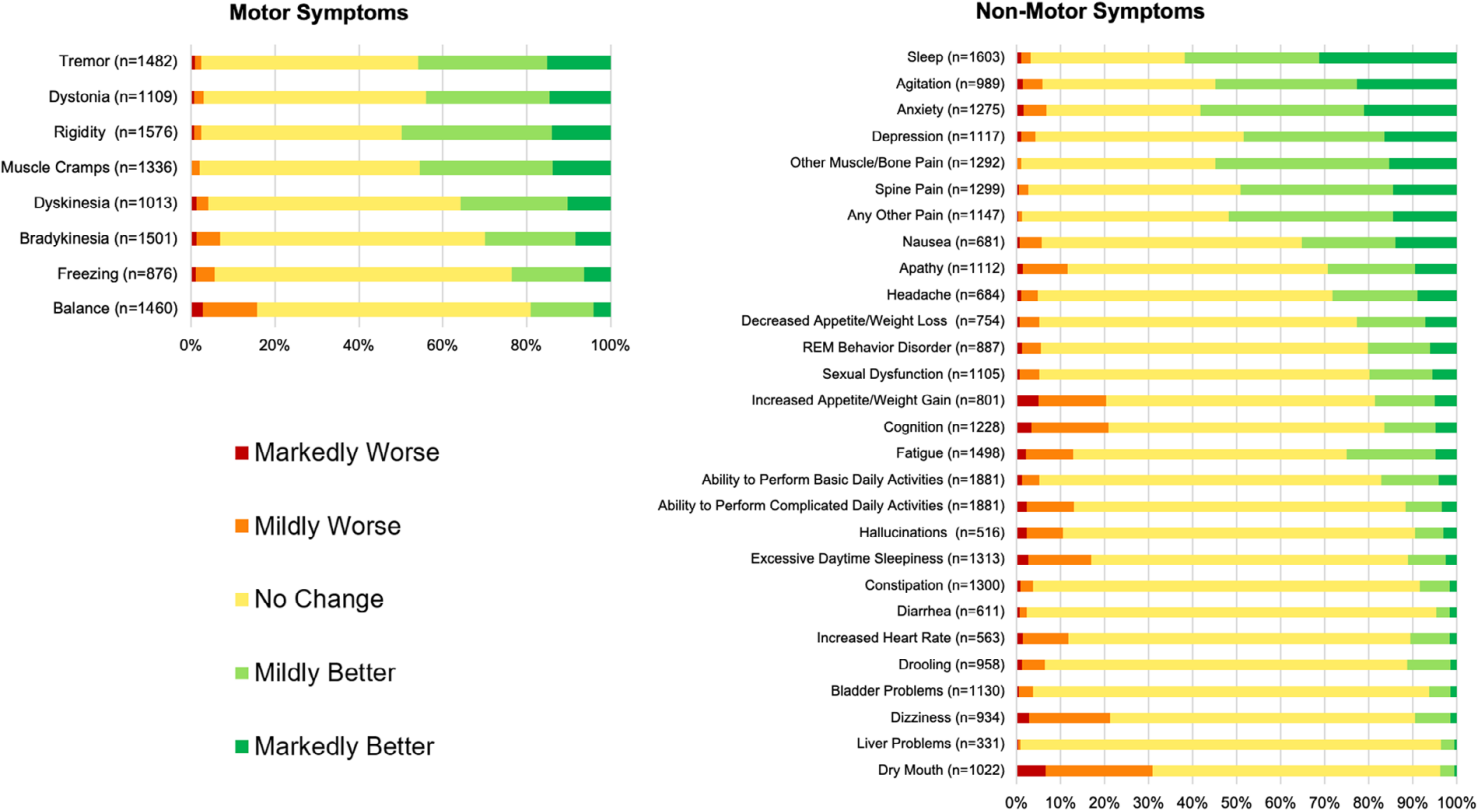
Self‐reported effects of cannabis use on pre‐existing Parkinson's disease symptoms. Frequency of responses ranging from markedly worse to markedly better with cannabis use for each of 36 pre‐existing motor and non‐motor symptoms among survey respondents with Parkinson's disease (n = number of respondents reporting that symptom prior to cannabis use).

Improvements in sleep, anxiety, agitation, and pain were the most commonly reported symptomatic benefits with cannabis use of any type, with more than 50% of respondents endorsing improvement (Fig. 1). Dry mouth, dizziness, cognitive impairment, increased appetite or weight, daytime sleepiness, imbalance, fatigue, palpitations, apathy and hallucinations were the most commonly reported adverse effects, with more than 10% of respondents endorsing worsening with cannabis use. The magnitude of the most frequent symptomatic effects was mild overall, ranging from 1.28 (pain, rigidity, bradykinesia, dyskinesias) to 1.51 (sleep) for improvements (Table 2), and from −1.12 (palpitations) to −1.25 (increased appetite or weight) for worsening (Table 3). Few respondents (<1.0%) reported the need to discontinue cannabis use due to worsening of any pre‐existing symptom or development of new bothersome symptoms.

**TABLE 2:** Magnitude of subjective symptomatic beneficial effects on pre‐existing Parkinson's disease symptoms with cannabis use (>25% reporting improvement)

Symptom	Number of respondents with this symptom	Magnitude of improvement *1 (mild) to 2 (marked)*	% with reported improvement	95% CI	
Sleep problems	1603	1.51	61.9%	59.5%	64.3%
Anxiety	1275	1.36	58.3%	55.6%	61.0%
Agitation	989	1.41	54.9%	51.8%	58.0%
Muscle or arthritis pain	1292	1.28	54.8%	52.1%	57.5%
Any other pain	1147	1.28	51.9%	49.0%	54.8%
Rigidity	1576	1.28	49.8%	47.3%	52.3%
Spine, low back or neck pain	1299	1.30	49.2%	46.5%	51.9%
Depression	1117	1.34	48.4%	45.5%	51.4%
Tremor	1482	1.33	46.0%	43.4%	48.5%
Muscle cramps	1336	1.30	45.5%	42.8%	48.2%
Dystonia	1109	1.33	43.9%	41.0%	46.8%
Dyskinesia	1013	1.28	35.7%	32.8%	38.7%
Nausea	681	1.40	35.2%	31.7%	38.8%
Bradykinesia	1501	1.28	29.9%	27.5%	32.2%
Apathy	1112	1.32	29.4%	26.7%	32.1%
Headache	684	1.32	28.2%	24.8%	31.6%

**TABLE 3:** Magnitude of subjective symptomatic adverse effects on pre‐existing Parkinson's disease symptoms with cannabis use (>5% reporting worsening)

Symptom	Number of respondents with this symptom	Magnitude of worsening *−1 (mild) to −2 (marked)*	% with reported worsening	95% CI	
Dry mouth	1022	−1.21	30.8%	28.0%	33.7%
Dizziness	934	−1.13	21.2%	18.6%	23.8%
Thinking or memory problem	1228	−1.16	20.9%	18.6%	23.1%
Increased appetite or weight	801	−1.25	20.2%	17.4%	23.0%
Daytime sleepiness	1313	−1.16	16.9%	14.9%	18.9%
Balance problems	1460	−1.17	15.7%	13.8%	17.6%
Ability to perform complicated ADLs	1881	−1.18	13.0%	11.5%	14.5%
Fatigue	1498	−1.17	12.8%	11.1%	14.4%
Increased heart rate	563	−1.12	11.7%	9.1%	14.4%
Apathy	1112	−1.13	11.5%	9.6%	13.4%
Hallucinations	516	−1.22	10.5%	7.8%	13.1%
Bradykinesia	1501	−1.19	6.9%	5.6%	8.2%
Anxiety	1275	−1.24	6.7%	5.3%	8.0%
Drooling	958	−1.18	6.4%	4.8%	7.9%
Agitation	989	−1.23	5.8%	4.3%	7.2%
Nausea	681	−1.13	5.6%	3.9%	7.3%
Freezing of gait	876	−1.18	5.6%	4.1%	7.1%
REM Behavior Disorder	887	−1.22	5.5%	4.0%	7.0%
Sexual dysfunction	1105	−1.14	5.2%	3.9%	6.5%
Ability to perform basic ADLs	1881	−1.23	5.2%	4.2%	6.2%
Decreased appetite or weight	754	−1.13	5.2%	3.6%	6.8%
Headache	684	−1.21	4.8%	3.2%	6.4%
Depression	1117	−1.23	4.2%	3.0%	5.4%
Dyskinesia	1013	−1.29	4.1%	2.8%	5.3%

**FIG. 2:**
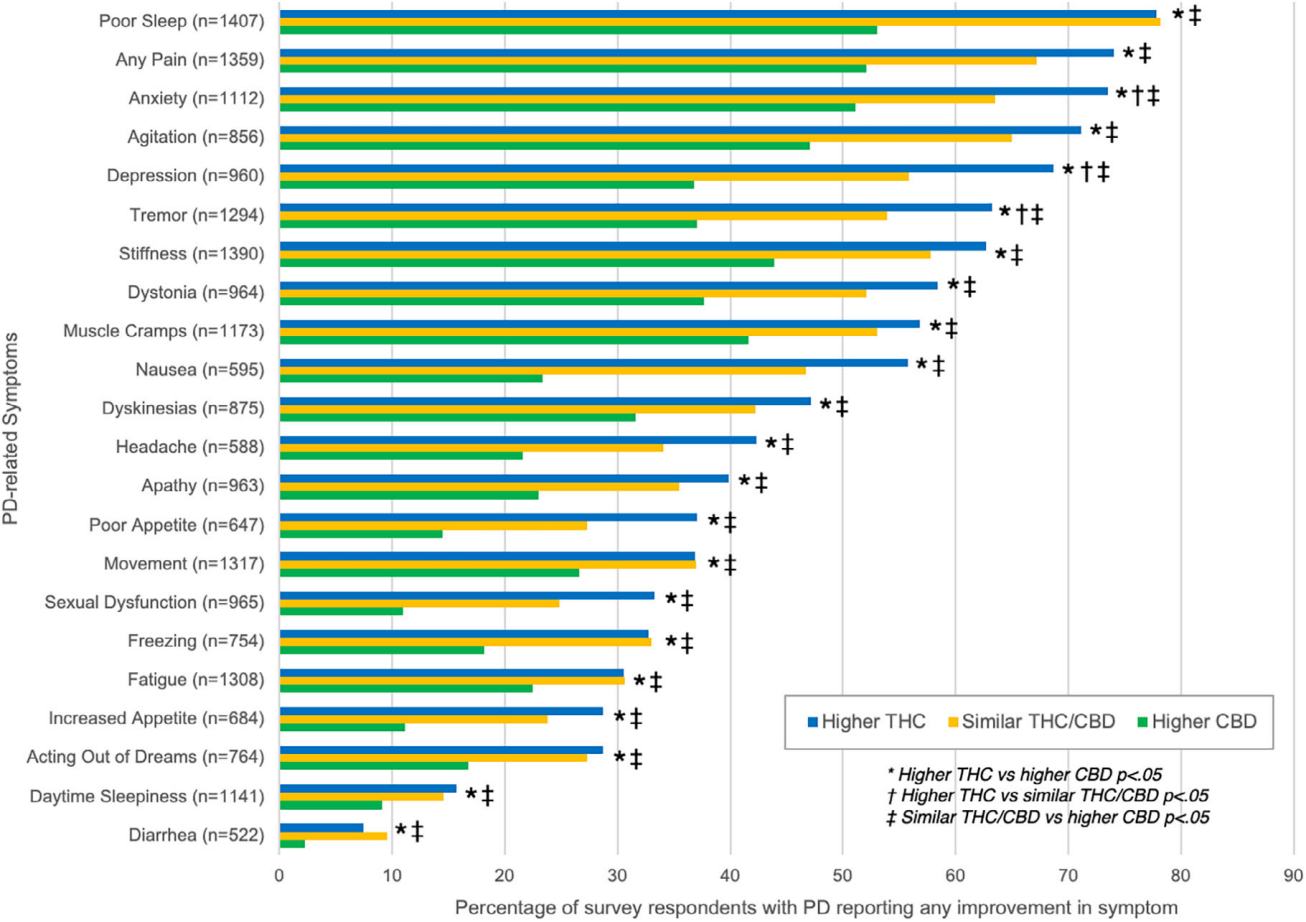
Differences in the frequency of reported symptomatic improvement between different cannabis product types. Higher THC product users (blue bars) reported symptomatic improvement at greater frequencies than higher CBD product users (green bars), with similar THC/CBD product users (yellow bars) generally reporting intermediate frequencies of improvement.

**FIG. 3:**
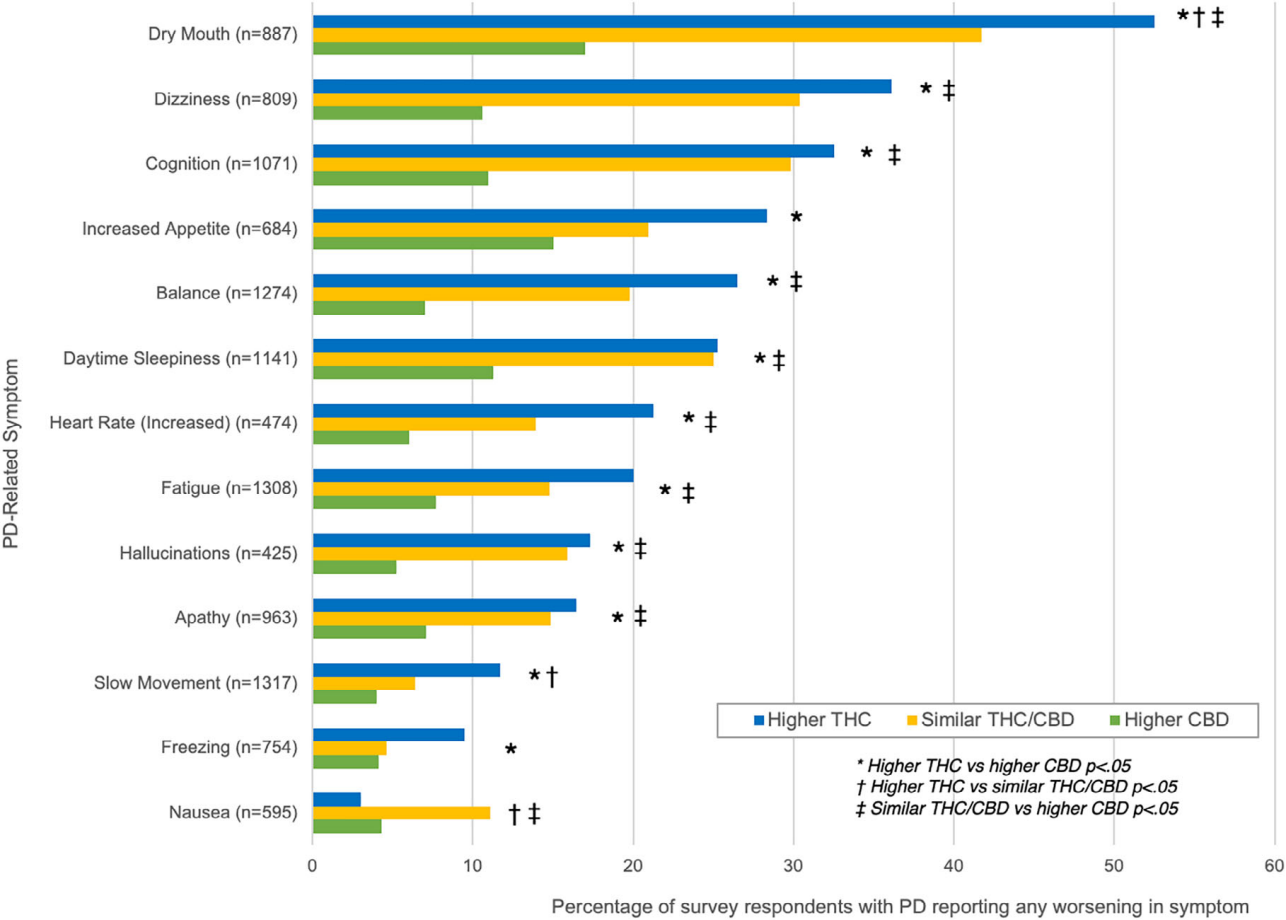
Differences in the frequency of reported symptomatic worsening between different cannabis product types. Higher THC product users (blue bars) reported symptomatic worsening at greater frequencies than higher CBD product users (green bars) except for worsening of nausea. Similar THC/CBD product users (yellow bars) reported intermediate frequencies of improvement overall.

There were significant groupwise differences in reported symptomatic effects (Fig. 2 and 3), both positive and negative, between “higher THC” (n = 493; 30.3% of respondents) and “higher CBD” (n = 890; 54.6%) product users. The “higher THC” group was more likely to report both improvement or worsening of pre‐existing PD‐related symptoms with cannabis use (all *P* < .01), except for worsening of nausea, which was not significantly different from “higher CBD” product users (*P* = 0.45).

The “similar THC/CBD” group (n = 247; 15.6%) generally reported symptomatic improvement and worsening at frequencies between those reported by the “higher THC” and “higher CBD” groups (Fig. 2 and 3). The “similar THC/CBD” group was significantly more likely to report improvements in all symptoms compared to the “higher CBD” group (all *P* < 0.05). There were no significant differences in reported symptomatic improvements between the “higher THC” and “similar THC/CBD” groups except for depression (OR 1.5, 95% CI 1.04, 2.07, *P* = 0.03), anxiety (OR 1.6, 95% CI 1.09, 2.0, *P* = 0.02), and tremor (OR 1.7, 95% CI 1.17, 2.57, *P* = 0.01), with “higher THC” more likely to improve than “similar THC/CBD”. Dry mouth (OR 1.5, 95% CI 1.02, 2.33, *P* = 0.04) and bradykinesia (OR 1.9, 95% CI 1.02, 3.68, *P* = 0.03) were more often reported to worsen with “higher THC” compared to “similar THC/CBD”. Nausea was more likely to worsen with “similar THC/CBD” than “higher THC” (OR 4.1, 95% CI 1.43, 11.55). Symptoms were more frequently reported to worsen in “similar THC/CBD” compared to “higher CBD” (all *P*< 0.03), except for freezing (*P* = 0.83), bradykinesia (*P* = 0.17), and increased appetite (*P* = 0.17).

When comparing effects on symptoms by specific dosages of cannabinoids, respondents taking >50 mg of THC (n = 72, 8.2%) reported improvements in constipation (*P* = 0.001) and diarrhea (*P* = 0.002) at significantly higher frequencies than those taking <50 mg of THC (n = 811, 91.9%). Regarding CBD dose, respondents taking >200 mg of CBD (n = 37, 3.67%) reported improvements in balance (*P* = 0.01), dyskinesia (*P* = 0.007), cognition (*P* = 0.02), and constipation (*P* = 0.002) at significantly higher frequencies than those taking <200 mg (n = 972, 96.3%). There were no significant differences in the frequency of reported worsening of any symptoms between the two THC dose groups (< or >50 mg) or between the two CBD dose groups (<or >200 mg).

### Impact of Cannabis Use on Prescription Medication Use

Respondents reported decreasing their prescription medication usage with cannabis use for pain (26.8%), anxiety (18.6%), sleep (16.9%), parkinsonism (13.9%), and depression (11.9%; Table 4). Fewer respondents reported discontinuing prescription medications for the treatment of a given symptom (<4.0%). There were differences in rates of reported changes in prescription medication use by cannabis product type, with “higher CBD” users less likely to report reduction in prescription medication usage compared to “higher THC” and “similar THC/CBD” (all *P*‐values <0.05; Table 5). There were greater magnitudes of differences in prescription medication decreases between the “higher CBD” and “higher THC” groups than between the “higher CBD” and “similar THC/CBD” groups.

**TABLE 4:** Reported impact of cannabis use on prescription medication use

Symptom being treated	*N*	Decreased, not stopped			Stopped		
		Decreased (%)	Lower 95% CI	Upper 95% CI	Stopped (%)	Lower 95% CI	Upper 95% CI
Pain	1226	26.8	24.4%	29.3%	3.7	2.6%	4.7%
Anxiety	1094	18.6	16.3%	20.9%	3.1	2.1%	4.1%
Sleep	1196	16.9	14.8%	19.0%	3.9	2.8%	5.0%
Tremor, slowness, stiffness	1594	13.9	12.2%	15.6%	1.8	1.1%	2.4%
Depression	1066	11.9	10.0%	13.9%	2.5	1.6%	3.5%

**TABLE 5:** Reported impact of THC and CBD product subtype use on prescription medication use

Symptom being treated	Decreased or stopped prescription medications (%)			Odds ratio (95% CI)		
	Higher THC	Similar THC/CBD	Higher CBD	Higher THC vs. similar THC/CBD	Higher THC vs. higher CBD	Higher CBD vs. similar THC/CBD
Pain	42.2%	38.1%	25.7%	1.19 (0.81, 1.75)	**2.11 (1.57, 2.83)**	**1.78 (1.24, 2.55)**
Anxiety	32.5%	32.2%	16.0%	1.01 (0.66, 1.55)	**2.53 (1.79, 3.56)**	**2.49 (1.64, 3.79)**
Sleep	30.3%	31.9%	15.7%	0.93 (0.62, 1.40)	**2.34 (1.68, 3.25)**	**2.52 (1.68, 3.78)**
Tremor, slowness, stiffness	22.5%	20.3%	12.0%	1.14 (0.76, 1.71)	**2.12 (1.54, 2.91)**	**1.86 (1.25, 2.78)**
Depression	21.5%	18.8%	11.5%	1.18 (0.71, 1.96)	**2.10 (1.41, 3.13)**	**1.79 (1.07, 2.98)**

Bold indicates *P*‐value <0.05.

## Discussion

Given increased interest in medical cannabis but paucity of scientific data supporting its efficacy for PD symptoms, our aim was to better understand patterns of real‐world cannabis use among people with PD, with particular interest in their subjective reports of its symptomatic effects. Along these lines, it is important to understand that cannabis legalization has led to the availability of a multitude of products with differing levels of cannabinoids and methods of ingestion. Thus, it is not simply a question of symptomatic effects, but rather how specific ratios of cannabinoids may impact PD‐related symptoms when consumed in a particular manner. To this end, we were able to survey 1881 people with PD who endorsed current or past use of cannabis post‐PD diagnosis.

Among our respondents, pre‐existing PD symptoms that most commonly improved with cannabis use included poor sleep, anxiety, agitation, and pain. Although only mild beneficial effects were reported, some respondents endorsed reduction in use of prescription medications when treating these symptoms with cannabis. It is unclear whether respondents reporting decreased prescription medication usage did so in consultation with their physicians, as 31% of respondents reported not discussing their cannabis use with their doctor. We therefore suggest that the subject of cannabis use be broached with patients with PD in a standardized, non‐judgmental manner during clinical encounters. Fortunately, the majority of people with PD using cannabis are not smoking or vaping and thus subjecting themselves to related pulmonary complications. Additionally, more respondents were using higher CBD products as opposed to THC products, perhaps attempting to minimize unwanted intoxicating effects.

Our survey results indicate potential differential symptomatic effects for higher THC versus higher CBD products. More specifically, use of higher THC products was associated with more frequently reported symptomatic benefits for pre‐existing PD‐related symptoms, especially for nausea, depression, tremor, poor sleep, agitation, decreased appetite, anxiety, sexual dysfunction, and pain. Higher THC users were also more likely to report reductions in prescription medications with cannabis use than higher CBD users. However, the likelihood of symptomatic benefit may not be the main driver in product selection for people with PD, with avoidance of unwanted side effects perhaps being more important. Along these lines, higher THC products were also associated with more frequent symptomatic worsening, most notably dry mouth, dizziness, cognition, and balance. Among people with PD, choice of cannabis product and its associated risk/benefit ratio judgment may be at least partially explained by self‐reported purpose of use, medicinal or recreational (or both). Over 80% of medicinal users reported using higher CBD products, whereas this pattern is reversed for recreational users, with over 60% reporting use of higher THC products. This finding mirrors that of a prior survey in multiple sclerosis.^22^ Presumably, medicinal users are seeking a beneficial effect on their symptoms but attempting to limit side effects, whereas recreational users may be seeking more intoxicating effects.

There could be a synergistic symptomatic effect between THC and CBD,^19^ with CBD potentially buffering the effects, both beneficial and adverse, of THC. The current findings largely support this, as the group using products with similar quantities of THC and CBD generally reported symptomatic improvement and worsening at frequencies between those reported by the higher THC and higher CBD product users. However, a notable exception is nearly equal frequencies of improved sleep among both the higher THC users and the group using products with similar quantities of THC and CBD. The literature on the effects of cannabis on sleep is complex and suggests that THC‐laden cannabis promotes sleep acutely, but tolerance occurs with chronic use, and withdrawal from chronic use impairs sleep. The effect of CBD is less clear—it may mitigate the sleep enhancing effect of THC, and addition of a small amount of THC to a CBD‐laden product may improve its effects on sleep.^23^ Previous studies in both animal models and humans suggest a biphasic dose response of cannabis, with differential therapeutic and/or intoxicating effects at lower and higher doses.^18^, ^20^, ^24^, ^25^, ^26^ However, there was no apparent dose‐dependent response on sleep when subdividing the THC group into higher and lower dose groups, nor when dividing the CBD group by dose. While our data do not support the biphasic model, this may be more related to the limitations of self‐reported doses and subjective effects than a true absence of biphasic responses in people with PD. In addition, our survey methods did not allow for evaluation of changes in cannabis product type or dosage within an individual, instead asking for the respondent to consider the product they were currently using or with which they had the most experience.

These results should be appreciated in the context of this study's limitations, especially given the bias inherent to survey data. There is some evidence of response bias, as respondents were younger, in earlier PD stages, and more likely to be college educated in comparison to the overall Fox Insight population who did not open the survey. However, differences are statistically significant only due to the large number of survey non‐participants within Fox Insight (n = 32,464), the effect sizes were small (e.g., age: 66.5 vs. 67.2), and responses were qualitatively similar. Also, while we could compare available demographics and clinical features of our respondents to both non‐respondents and non‐finishers of the survey, there are additional characteristics that could have contributed to the decision to respond that are not captured by Fox Insight. For example, it is unclear how the past experiences with cannabis, good or bad, may have influenced their decision to complete the survey, as well as how to respond. However, it is noted that cannabis was rated as having “no effect” on many symptoms overall, tempering the concern that responses were biased in either direction. A further concern is that some responses are based on infrequent cannabis use, with 15.2% of respondents using cannabis less than monthly, and 21.5% using it for less than one month. While limited experience with cannabis may have affected respondents' survey responses, we included all responses to improve generalizability of results at this early stage of research.

Despite these limitations, our analyses of the symptomatic impact of more specific cannabis product types (i.e., higher THC or higher CBD products) may serve as a guide to future randomized‐controlled trials and product selection based on the PD‐related symptom of interest. This position is bolstered by the fact that almost 87% of respondents reported the THC:CBD ratio in their product and more than two‐thirds knew exact dosages. For example, a clinical trial focused on improving sleep in PD may choose to test a product with similar amounts of THC and CBD, given similar frequency of symptomatic improvement to the higher THC group with lower rates of adverse effects overall, based on our data. However, it may be premature to utilize the results of this study to counsel individual patients with PD regarding specific aspects of cannabis use. Labeling of THC and CBD content in products may be inaccurate,^27^ and there are limitations inherent in the process of self‐reporting of product type and dosage given. Further, reports of symptom improvement/worsening are retrospective and there are other potential moderating factors (e.g., length of time of use, concomitant medications) that must be considered. Cannabis products contain many chemicals other than cannabinoids, such as terpenes and flavonoids, that may contribute to symptomatic effects, and it was not possible to ascertain this information within the confines of this survey.

In summary, people with PD report that cannabis subjectively improves some PD‐related symptoms, with higher THC products conferring more frequent benefits than higher CBD products. However, with greater reward there is also greater risk, as higher THC products were associated with more adverse effects. Cannabis product choice among people with PD may be more predicated on avoidance of unwanted side effects, especially for those self‐classifying as medicinal users. These survey results offer a broad overview of real‐world cannabis use patterns and experience among a large group of people living with PD and provide initial results regarding the differential symptomatic effects of higher THC versus higher CBD products. Next steps should include more rigorous, controlled studies, informed by the results herein, to more objectively study the effects of varying types of cannabis on PD symptoms, as well the impact of the different methods of ingestion and specific doses.

## Author Roles

(1) Research project: A. Conception, B. Organization, C. Execution; (2) Statistical Analysis: A. Design, B. Execution, C. Review and Critique; (3) Manuscript: A. Writing of the first draft, B. Review and Critique.

SKH: 1A, 1B, 1C, 2C, 3A, 3B.

CD: 1A, 1B, 1C, 2C, 3A, 3B.

SS: 1A, 2A, 2B, 2C, 3A, 3B.

YL: 1A, 1B, 1C, 2C, 3A, 3B.

ML: 1A, 1B, 1C, 2C, 3A, 3B.

## Disclosures

**Ethical Compliance Statement:** This study was approved by the New England IRB (IRB # 120160179, 183451.0), in accordance with all applicable national and/or international guidelines/laws.

**Funding Sources and Conflicts of Interest:** This study was funded by the Michael J. Fox Foundation for Parkinson's Research (Grant # 18255). There were no conflicts of interest for any authors.

**Financial Disclosures for the Previous 12 Months:** Dr. Holden has received grant funding from the Michael J. Fox Foundation for Parkinson's Research and a loan repayment award from the National Institute of Neurological Disorders and Stroke (L30 NS103315). Dr. Leehey has received grant funding from the Parkinson's Progression Markers Initiatives, Neuraly Inc., the Michael J. Fox Foundation, the NIH, the Colorado Department of Public Health and Environment, Biogen, US WorldMeds LLC, and Vtesse. All other authors have no disclosures to report.

## Supporting information


**Supplementary Text A:** Fox Insight Survey Email Invitations. Three Emails were sent to Fox Insight participants: (1) Initial invitation announcing the survey. (2) Second invitation announcing that the survey can now be completed on a mobile phone or tablet. (3) Third invitation announcing there was only three days left to complete the survey.

**Supplementary Text B:** Fox Insight Survey. (1) Survey Introduction. The Introduction to the survey provided definitions of cannabis, cannabis constituents, and types of cannabis products; and asked respondent to fill out the survey regarding the one type of cannabis product that they took the most. (2) Survey Questions. The 15 questions that were provided in electronic format.
