# Supplementary material for: Higher Risk, Higher Reward? Self‐Reported Effects of Real‐World Cannabis Use in Parkinson's Disease
Source: Mov Disord Clin Pract. 2022 Jan 28;9(3):340–50. doi: 10.1002/mdc3.13414 (PMC8974868; doi:10.1002/mdc3.13414)
Supplement: Supplementary file 1 — Supplementary Text A: Fox Insight Survey Email Invitations. Three Emails were sent to Fox Insight participants: (1) Initial invitation announcing the survey. (2) Second invitation announcing that the survey can now be completed on a mobile phone or tablet. (3) Third invitation announcing there was only three days left to complete the survey. [file MDC3-9-340-s001.docx]

| Subject | Share Your Experience with Cannabis Use to Treat Parkinson’s |
| --- | --- |
| Title | Invitation::Cannabis Use in PD |
| Body | In this Fox Insight survey, we would like to understand how people with Parkinson’s disease may use marijuana (cannabis) to treat symptoms. Because there are many forms of cannabis available, it can be challenging to study and measure. This means there is limited research on this topic. Your answers to the survey could help researchers better understand the use and effects of cannabis among people with Parkinson’s and to help support future research in this area.  The questionnaire should take between 15 and 20 minutes to complete, depending upon the amount and type of cannabis use. Completing the survey is optional and will not affect your enrollment in Fox Insight. The privacy and confidentiality of study participants is always a primary concern; additional legal protection has been secured to safeguard respondents and the information they provide as a part of this survey. |
| Call to Action Text | Take the survey now! |
| Call to Action URL | https://foxinsight.michaeljfox.org/surveys |
| Signature | The study is sponsored by The Michael J. Fox Foundation. If you have any questions about the study, please contact [info@foxinsight.org](mailto:info@foxinsight.org). Thank you for participating in Fox Insight and powering Parkinson’s research.   Best, The Fox Insight Study Team |

1. Initial invitation announcing the survey

| Subject | Reminder: Share Your Experience with Cannabis Use to Treat Parkinson’s |
| --- | --- |
| Title | Reminder::Cannabis Use in PD v2 |
| Body | Recently, we reached out to you to take part in a survey in Fox Insight exploring the use and effects of marijuana (cannabis) among people with Parkinson’s disease (PD).  This survey has been expanded so mobile and tablet users in addition to desktop/laptop users can complete the survey.  For the best experience, we recommend completion on a desktop or laptop.  If you have taken cannabis since being diagnosed with PD, we want to hear from you! In this survey, we would like to learn how you may be using cannabis to treat Parkinson’s symptoms. With your help, we could better understand the effects of cannabis and help support future research in this area.  Completing the survey is optional and will not affect your enrollment in Fox Insight. The privacy and confidentiality of study participants is always a primary concern; additional legal protection has been secured to safeguard respondents and the information they provide as a part of this survey.  **If you have taken cannabis since being diagnosed with PD, please click on the link below.** |
| Call to Action Text | Take the survey now! |
| Call to Action URL | https://foxinsight.michaeljfox.org/surveys |
| Signature | We sincerely appreciate your contribution to this important project. If you have any questions about the study, please contact [info@foxinsight.org](mailto:info@foxinsight.org).  Thank you for participating in Fox Insight and powering Parkinson’s research.  Best, The Fox Insight Study Team |

1. Second invitation announcing that the survey can now be completed on a mobile phone or tablet

| Subject | Reminder: Share Your Experience with Cannabis Use to Treat Parkinson’s |
| --- | --- |
| Title | Reminder::Cannabis Use in PD v2 (3 Day) |
| Body | Recently, we reached out to you to take part in a survey in Fox Insight exploring the use and effects of marijuana (cannabis) among people with Parkinson’s disease (PD).  This survey has been expanded so mobile and tablet users in addition to desktop/laptop users can complete the survey.  For the best experience, we recommend completion on a desktop or laptop.  If you have taken cannabis since being diagnosed with PD, we want to hear from you!  In this survey, we would like to learn how you may be using cannabis to treat Parkinson’s symptoms. With your help, we could better understand the effects of cannabis and help support future research in this area.  Completing the survey is optional and will not affect your enrollment in Fox Insight.  The privacy and confidentiality of study participants is always a primary concern; additional legal protection has been secured to safeguard respondents and the information they provide as a part of this survey.  **If you have taken cannabis since being diagnosed with PD, please click on the link below.** |
| Call to Action Text | Take the survey now! There are three days left to participate. |
| Call to Action URL | https://foxinsight.michaeljfox.org/surveys |
| Signature | We sincerely appreciate your contribution to this important project.  If you have any questions about the study, please contact [info@foxinsight.org](mailto:info@foxinsight.org).  Thank you for participating in Fox Insight and powering Parkinson’s research.  Best, The Fox Insight Study Team |

1. Third invitation announcing there was only three days left to complete the survey.
