# Supplementary material for: Higher Risk, Higher Reward? Self‐Reported Effects of Real‐World Cannabis Use in Parkinson's Disease
Source: Mov Disord Clin Pract. 2022 Jan 28;9(3):340–50. doi: 10.1002/mdc3.13414 (PMC8974868; doi:10.1002/mdc3.13414)
Supplement: Supplementary file 2 — Supplementary Text B: Fox Insight Survey. (1) Survey Introduction. The Introduction to the survey provided definitions of cannabis, cannabis constituents, and types of cannabis products; and asked respondent to fill out the survey regarding the one type of cannabis product that they took the most. (2) Survey Questions. The 15 questions that were provided in electronic format. [file MDC3-9-340-s002.docx]

**Supplementary Text B: Fox Insight Survey**

1. **Survey Introduction**

Cannabis (marijuana) is made up of many chemicals, but the research team is specifically interested in:

Tetrahydrocannabinol (THC) - makes people feel “high” (euphoria, intoxicated, etc.)

Cannabidiol (CBD) – does not make people feel “high”

Cannabis products can be classified according to the relative amounts of THC and CBD in them. We would like to know which type of cannabis product with which you have the most experience, as well as the benefits and side effects you experienced with this product. If you are taking cannabis, please look at the package or label to decide which type it is.

**High CBD, low THC** - is usually purchased from a dispensary and is supposed to give you more effects from the CBD than the THC, so that you do not get intoxicated, or “high”. This is different from hemp because hemp has <0.3% THC. Products in this category also include CBD oil if purchased at a cannabis dispensary. The product label may say “cannabis-infused”.

**High THC, low/no/unknown CBD** – is usually purchased from a dispensary and is supposed to have enough THC to cause you to feel some “high”. The product label may say “cannabis-infused”.

**Similar amounts of THC and CBD** - is usually purchased from a dispensary and is supposed to give you more mild effects of THC along with CBD, with less of a “high”. The product label may say “cannabis-infused”.

**Hemp** - is from a cannabis plant, is high in CBD and is supposed to have <0.3% THC. Because it has such low THC it is sold outside of cannabis dispensaries: online and in a variety of stores. This category includes many CBD oil products. The product may not even have CBD anywhere on the label, but if it is hemp then it is mostly CBD, and the mg of hemp, for the purposes of this survey, is the same as the mg of CBD. The product label may say “CBD-infused” or “hemp-infused.

Usually cannabis products with >0.3% THC are bought from a dispensary, but some people grow their own cannabis plants, and some may get cannabis from unsanctioned sources.

We ask you fill out this survey regarding the type of cannabis (marijuana) product you have used THE MOST. Even if you do not know which type you taken, we are still interested in your experience.

1. **Survey Questions**

Q1. Who is filling out this survey?

1. Person with PD
2. Caregiver (On Behalf of Person with PD)
3. Person with PD and Caregiver in Equal Proportion

Q2. Are you filling out this survey about cannabis you are taking now or have taken in the past?

1. Now
2. Not now, but < 1 year ago
3. > 1 year ago

Q3. What type of cannabis do/did you use most frequently (select all that apply)?

1. High CBD/low THC
2. High THC/low CBD
3. Similar amounts of CBD and THC
4. Hemp
5. All of the above
6. Don’t know

Q4. If you use/used particular cannabis (marijuana) products, please list their brand name(s) here:

1. List their brand names here:
2. Don’t know the brand name

Q5. On the days you take/took THC products, how much THC do/did you take total per day on average?

1. up to 5 mg
2. 6 - 50mg
3. > 50 mg
4. Don’t know
5. Not applicable, because I use/used hemp

Q6. On the days you take/took CBD products, how much CBD do/did you take total per day on average?

1. Up to 5 mg
2. 6 - 50mg
3. 51 - 200 mg
4. 201 - 600 mg
5. >600 mg
6. Don’t know

Q7. How do you know how much CBD and THC is in the cannabis product that you took? (check all that apply)

1. A dispensary worker told me the product had more or less of CBD compared to THC
2. I have the package and the amounts are on the label
3. I don’t know how much I took
4. Other:

Q8. How do/did you usually take this cannabis product? (check all that apply)

1. Smoke/combustion (cigarette, pipe)
2. Vape (Vaporizer/Vape pen)
3. Swallow: food (edible: gummy, chocolate bar, etc.)
4. Swallow: drink (beverage)
5. Swallow: oil
6. Sublingual/Tincture
7. Apply to skin (oil, lotion, cream)
8. Patch
9. Suppository

Q9. How often do/did you take this type of cannabis?

1. less than once a month
2. less than 1 time a week, but at least once/month
3. not daily, but more than 1 time/week
4. 1 time a day
5. 2-3 times a day
6. more than 3 times a day

Q10. How long have you taken or did you take this type of cannabis, total?

1. < 1 month
2. 1 - 6 months
3. 7 months – 1 year
4. > 1 year

Q11. Please check the effect that this type of cannabis has/had on:

Worst <----------------------No effect ---------------------> Best

| I have NOT HAD this symptom | This symptom STARTED WITH cannabis | Had symptom; cannabis made it MARKEDLY WORSE | Had symptom; cannabis made it MILDLY WORSE | Had symptom; cannabis had NO EFFECT on this symptom | Had symptom; cannabis made it MILDLY BETTER | Had symptom; cannabis made it MARKEDLY BETTER |
| --- | --- | --- | --- | --- | --- | --- |

1. Tremor
2. Slow movement in general
3. Stiffness
4. Balance problems
5. Dyskinesia (extra movement from levodopa)
6. Dystonia (sustained muscle contraction of foot/leg or hand/arm)
7. Muscle cramps
8. Freezing (your foot or feet won’t move, usually occurs when you start to walk or when turning)
9. Thinking or memory problem
10. Hallucinations
11. Depression
12. Anxiety
13. Agitation
14. Apathy (lack of interest or motivation)
15. Fatigue
16. Sleep problems at night
17. Acting out dreams
18. Daytime sleepiness
19. Spine pain, low back or neck
20. Muscle or arthritis pain (other than spine pain)
21. Any other pain
22. Headache
23. Bladder problems
24. Sexual dysfunction
25. Constipation
26. Diarrhea
27. Nausea
28. Dizziness
29. Decreased appetite or weight
30. Increased appetite or weight
31. Liver problems
32. Dry mouth
33. Drooling
34. Increased heart rate
35. Ability to perform complicated daily activities (paying bills, cooking, driving)
36. Ability to perform basic daily activities (dressing, grooming)
37. Other _________________

Q12. Have you taken a different type of cannabis in the past and stopped it due to negative side effects?

1. Yes
2. No

Q13. Have you discussed the use of cannabis with any of your physicians?

1. Yes
2. No

Q14. What would you consider to be the primary purpose of your cannabis use?

1. Medicinal
2. Recreational
3. Both

Q15. Do you feel that using cannabis has caused you to change how much prescription medication you take for the below conditions?

|  | No change | Increased | Decreased | Stopped | Not applicable (not taking any prescription medication for this) |
| --- | --- | --- | --- | --- | --- |
| Hallucinations |  |  |  |  |  |
| Tremor, slowness, stiffness |  |  |  |  |  |
| Pain |  |  |  |  |  |
| Sleep |  |  |  |  |  |
| Anxiety |  |  |  |  |  |
| Depression |  |  |  |  |  |

MJFF is collaborating with the Parkinson’s Foundation to explore related research questions in this area. Have you recently completed a survey on Cannabis use and Parkinson’s, distributed by the Parkinson’s Foundation (or PF representative)?

1. Yes
2. No

This is the last page of the survey. If you want to review or change any of your answers please click the back arrow below. If you are satisfied with all your answers click the forward arrow below and you will have submitted your answers and see a summary of them.
